# Knowledge, attitudes, and practices regarding asthma management among pharmacists in palestine: A cross-sectional study

**DOI:** 10.1371/journal.pone.0351933

**Published:** 2026-06-18

**Authors:** Dima Masri, Haneen Joulani, Alisse Nasser, Dala N. Daraghmeh

**Affiliations:** 1 Pharmaceutical Research and Innovation Hub, Department of Pharmacology, Faculty of Pharmacy, Al-Quds University, Jerusalem, Palestine; 2 Pharmacy Department, Faculty of Pharmacy, Nursing and Health Professions, Birzeit University, Birzeit, State of Palestine; Yarmouk University, JORDAN

## Abstract

**Background:**

Pharmacists can play a key role in asthma management through patient education, inhaler technique assessment, and promoting evidence-based care. This study assessed Palestinian pharmacists’ general asthma knowledge, asthma pharmaceutical care knowledge, attitudes, practices, and perceived barriers to asthma management.

**Methods:**

A cross-sectional study was conducted from March to June 2025 in Palestine. Data were collected using a self-administered online questionnaire (convenience/volunteer-response sampling; response rate not determinable). General asthma knowledge was scored by awarding 1 point per correct response (range 0–17) and categorized as poor (0–8), fair (9–11), and knowledgeable (12–17). Data were analyzed using SPSS, bivariate associations were tested using chi-square test, or Fisher’s exact test, and multivariable linear regression was used to identify independent predictors. A p-value of < 0.05 was considered statistically significant.

**Results:**

A total of 402 participants (137 male and 265 female) participated in this study. Overall, 57.2% were classified as knowledgeable in general asthma knowledge. Pharmacists working in hospital settings showed a significantly higher asthma knowledge in comparison with those working in community pharmacies (β = 0.110, p = 0.032). However, the model explained minimal variance (R² = 0.037) and was not statistically significant overall (overall model p = 0.144). No pharmacists’ characteristics were significantly associated with asthma pharmaceutical care knowledge (R² = 0.027; overall model p = 0.378) or attitudes and practices (R² = 0.014; overall model p = 0.850).

**Conclusion:**

Pharmacists demonstrated mixed asthma knowledge with gaps relevant to updated guideline-based care, alongside practice barriers and clinically important practice gaps. Given the very low R² values, observed associations should be interpreted cautiously. Structured continuing professional development and targeted training may improve pharmacists’ uptake of updated recommendations and strengthen asthma pharmaceutical services.

## Introduction

Asthma is a chronic inflammatory ‌‌airway disease characterized by variable airflow limitation and respiratory symptoms such as wheezing, shortness of breath, chest tightness, and cough. It affects both children and adults and remains a major global public health problem [1]. Physician-diagnosed asthma has been estimated at 3.8% in Palestine, and about 300 million people worldwide live with asthma, causing approximately 1000 deaths per day, especially in low-middle-income countries [2]. Underdiagnosis and undertreatment contribute to this burden. Undertreated asthmatics report sleep disturbances, poor concentration, daytime fatigue, and school or work absenteeism, which collectively reduce quality of life and impose a substantial economic burden on patients and the health system [3]. Recurrent exacerbations often result in emergency department visits and hospital admissions, and in severe cases, may lead to preventable deaths [3,4].

Poor control frequently stems from modifiable factors like chronic exposure to triggers, non-adherence to treatment, inappropriate inhaler technique, and insufficient implementation of prescribed therapies [1]. Proper inhaler technique is essential for asthma treatment to reach the lower airways and thus be effective, and numerous studies indicate widespread inhaler misuse [5]. Recent asthma guidelines therefore emphasize the need to implement structured patient education, regular monitoring, and the use of evidence-based treatment regimens within a multidisciplinary model of care [1,6].

Pharmacists are highly accessible, assist in managing chronic conditions like asthma, and their role has evolved from medication dispenser to clinical practitioner who provides individualized patient care based on their clinical expertise [7]. Additionally, drug-related problems (DRPs), which are events or conditions resulting from drug therapy that hinder the achievement of desired health outcomes, are a major concern for treatment outcome [1]. To reduce DRPs and enhance medication adherence, asthma knowledge, and inhaler technique, pharmacists should be integrated into the healthcare system to provide pharmaceutical care (PC) services and optimize medication use [4,8]. Previous studies indicated that pharmacists’ pharmaceutical care services improve patients’ health by identifying, preventing, and resolving drug-related issues and encouraging proper medication use [1,4]. Despite the international Global Initiative for Asthma (GINA) guideline, which aims to optimize asthma care and management, asthma morbidity and mortality remain alarmingly high, and many asthma deaths still result from preventable factors like inadequate treatment and monitoring [9].

Within this context, a Knowledge–Attitudes–Practices (KAP) framework helps structure pharmacists’ roles in asthma care: knowledge reflects guideline-based understanding, attitudes indicate readiness and perceived responsibility, and practices represent delivered care behaviors; individual and system barriers may hinder translation into practice.

Although asthma-related KAP has been studied internationally, evidence from Palestine remains limited, particularly regarding updated guideline knowledge, pharmaceutical care competencies, and barriers contributing to gaps between knowledge and practice.

The adherence of healthcare professionals, including pharmacists, to international guideline recommendations is inconsistent. Therefore, this study, to the best of our knowledge represents one of the first studies in Palestine to comprehensively assess asthma-related KAP among pharmacists, aims to assess pharmacists’ general asthma knowledge, asthma pharmaceutical care knowledge, attitudes, practices, and perceived barriers related to asthma management, and to explore factors associated with better performance.

## Methods

### Study design

A cross-sectional study was carried out over a period of 4 months from March 2025 to June 2025 across Palestine.

### Study population, sampling procedure and sample size calculation

The target population included licensed pharmacists currently practicing in Palestine. Participants were included if they were currently practicing in a community pharmacy, or hospital pharmacy settings and provided informed consent before accessing the survey. Pharmacists who were retired, not currently practicing in Palestine, working overseas, or those who refused to participate were excluded. Participants were recruited using a non-probability, convenience (volunteer-response) online sampling approach. Because the survey link was disseminated through open channels without a fixed denominator, a response rate could not be calculated. This sampling approach may introduce selection bias and limits generalizability.

An automated software program, the Raosoft calculator for sample size (http://www.raosoft.com/samplesize.html), was used to calculate the required sample size for this study. The minimum target sample size was 361, with a 5% margin of error and a 95% confidence level. A population size of 5,932 registered pharmacists in Palestine was used (Palestinian Pharmacy Association, 2023). The target sample size was increased by 5% to 10% to account for incomplete responses and improve precision.

### Data collection instrument

Data was collected using a self-administered questionnaire in the native Arabic language, originally written in English and then translated into Arabic. This questionnaire was created following a thorough review of the literature [1,6] and study aims and objectives. The initial draft underwent evaluations for content and validity. The validity check occurred in two stages: first, a panel of academicians familiar with questionnaire design and clinical and community pharmacists assessed the questionnaire for content validity. Based on their feedback and suggestions, the questionnaire was revised. In the second stage, a pilot study was conducted with an updated survey. The survey was piloted with a group of 20 people to improve the clarity and readability of the survey items, as well as to ensure their relevance to the intended target population. Following the pilot study, further adjustments were considered. Pilot data was excluded from the final analysis.

### Reliability and validity test results

Content Validity was assessed using the content validity Index (CVI), which was 0.81, indicating acceptable content validity. Item-level CVI (I-CVI) values were not available because item-level expert ratings were not retained; this is acknowledged as a limitation. Internal consistency reliability was evaluated using domain-appropriate reliability coefficients (Table 1). Because the pharmacists’ general knowledge items were dichotomously scored (correct/incorrect), reliability for this section was assessed using Kuder–Richardson Formula 20 (KR-20 = 0.75). The asthma pharmaceutical care knowledge section (9 items) showed good internal consistency (α = 0.81), the pharmacist attitudes and behaviors section (12 items) demonstrated excellent internal consistency (α = 0.87). Exploratory factor analysis was not conducted; therefore, construct validity of multi-item domains was not formally evaluated and is acknowledged as a limitation.

**Table 1:** Reliability coefficient of the questionnaire.

Section	No. of items	Reliability coefficient
Pharmacists’ general knowledge	17	KR-20 = 0.75
Asthma pharmaceutical care knowledge’	9	Cronbach’s Alpha = 0.81
Pharmacist attitudes and behaviors	12	Cronbach’s Alpha = 0.87

KR-20 was used for the dichotomously scored knowledge items (correct/incorrect), while Cronbach’s alpha was used for Likert-type domains.

### Survey content

The final questionnaire (S1 File) contains seven sections, each with a specific number of questions as follows:

- **The first section** includes questions on sociodemographic characteristics, such as gender, age group, professional role, educational level, years of experience, working hours per week, geographic location, and province.
- **The second section** assesses pharmacists’ knowledge regarding their familiarity with asthma concepts. Responses are based on multiple choice, multiple selection, and either “correct” or “incorrect”.
- **The third section** is asthma pharmaceutical care knowledge, which refers to the understanding and application of information related to the management of asthma. Responses are based on multiple choice, multiple selection, and “agree,” “neutral,” or “disagree.”
- **The fourth section** assesses attitudes and practices towards recommending asthma treatment, such as reasons for advising or not advising, situations for which drugs are recommended, and barriers to prescribing them. Responses are based on answers that allow for multiple selections.
- **The fifth section,** which consists of four questions, assesses the pharmacist’s access to accurate, up-to-date, and evidence-based information regarding asthma management
- **The sixth section** addresses the personal consumption of asthma medication, including whether the health care workers have themselves used medicines and the sources from which they acquired them.
- **The seventh section** addresses the challenges and barriers; this section contains 12 yes or no questions.

Some knowledge items were simplified for survey feasibility and may not fully capture clinically nuanced concepts. Items related to infection control and pharmacotherapy were included to reflect both core asthma knowledge and contextual clinical decision-making considerations.

### Survey distribution and administration

The questionnaire was distributed using a Google Forms platform from March 2025 to June 2025. Eligible participants were provided the survey link via email, WhatsApp, and SMS. Numerous methods were employed to locate and recruit qualified participants, including direct approach by the research team and dissemination of the survey link via social media platforms. Recruitment was conducted through professional networks and online channels; therefore, the sampling frame was not restricted to a single list and the total number of pharmacists who received or viewed the link was not measurable.

### Statistical analysis

After data collection, the data were extracted and recorded in an Excel workbook (Microsoft Office, 2013). Prior to the analysis, data cleaning, coding, and grouping were conducted. Data was summarized using descriptive statistics. All variables collected throughout the questionnaire were presented by calculating the frequency (%) for binary variables and the mean ± standard deviation (SD) for continuous variables. The general asthma knowledge score was calculated by assigning one point to each correct response (range 0–17) and was additionally categorized as poor (0–8; ≤ 50%), fair (9–11; 51–69%), and knowledgeable (12–17; ≥ 70%), based on commonly used thresholds in KAP studies to facilitate interpretation and comparison with existing literature. Similar categorization approaches have been applied in previous studies assessing healthcare professionals’ knowledge, allowing comparability across studies [10,11]. These categories were used for descriptive purposes only, while the continuous knowledge score was used in all inferential analyses.

Bivariate associations between pharmacists’ characteristics and knowledge categories were assessed using chi-square test or Fisher’s exact tests, as appropriate. To identify independent predictors of knowledge scores, multivariable linear regression models were fitted using the continuous knowledge score as the dependent variable and sociodemographic and workplace variable as predictors, standardised beta coefficients (β) and p-values were reported. Regression diagnostics were performed to assess model assumptions (linearity/specification, residual normality, homoscedasticity, and influential observations). Multicollinearity was assessed using generalized variance inflation factors (GVIF). For variables with multiple degrees of freedom, adjusted GVIF values (GVIF^(1/(2 × Df))) were interpreted. The adjusted GVIF values ranged from 1.04 to 1.47, indicating no evidence of problematic multicollinearity.

Given the bounded nature of the score, a sensitivity analysis was conducted using Poisson regression based on the original count form of the knowledge score (0–17). This analysis yielded results consistent with those of the linear regression model. A two-sided p-value <0.05 was considered statistically significant. All analyses were conducted using SPSS version 26.

### Ethical consideration

The study protocol was approved by the Research Ethical Committee (REC) of Al-Quds University (archived number: 511/REC/2025). Prior to each participant’s participation in the study, informed consent was obtained, and respondents were informed about the questionnaire content. Participants’ information and data were coded, and privacy was highly considered. All methods were performed in accordance with the relevant guidelines and regulations

## Results

### Sociodemographic characteristics and pharmacists’ asthma management knowledge

A total of 402 participants participated in this study. Due to the various methods used to identify and recruit eligible participants, such as social media, determining the response rate was not feasible. Table 2 presents the sociodemographic characteristics of participants by knowledge score. The majority were females (65.9%), aged 20–29 (59.2%), staff pharmacists (79.4%) and worked in community pharmacies (89.1%). Additionally, 77.4% of participants had a bachelor of pharmacy degree, 31.8% had less than a year of experience, and 43.3% worked 25–40 hours per week. S1 Table.

**Table 2:** Sociodemographic data distribution and pharmacists’ asthma management knowledge.

Variable	Category	Total (%)	Poor N (%)	Fair N (%0	Knowledgeable N (%)	P-value
Gender	Male	137 (34.1)	13 (9.5)	36 (26.3)	88 (64.2)	0.088
	Female	265 (65.9)	41 (15.5)	82 (30.9)	142 (53.6)	
Age (years)	20-29	238 (59.2)	31 (13)	74 (31.1)	133 (55.9)	0.604
	30-39	80 (19.9)	15 (18.8)	22 (27.5)	43 (53.8)	
	40-49	55 (13.7)	6 (10.9)	14 (25.5)	35 (63.6)	
	≥50	29 (7.2)	2 (6.9)	8 (27.6)	19 (65.5)	
Profile	Manager	49 (12.2)	2 (4.1)	11 (22.4)	36 (73.5)	0.014*
	Owner	34 (8.5)	5 (14.7)	16 (47.1)	13 (38.2)	
	Staff pharmacist	319 (79.4)	47 (14.7)	91 (28.5)	181 (56.7)	
Working setting	Community pharmacy	358 (89.1)	50 (14)	110 (30.7)	198 (55.3)	0.241
	Hospital pharmacy outpatient	16 (4.0)	1 (6.3)	4 (25)	11 (68.8)	
	Hospital pharmacy inpatient	28 (7.0)	3 (10.7)	4 (14.3)	21 (75)	
Educational level	Bachelor of Pharmacy	311 (77.4)	45 (14.5)	96 (30.9)	170 (54.7)	0.163
	PharmD	37 (9.2)	3 (8.1)	7 (18.9)	27 (73)	
	Master	48 (11.9)	6 (12.5)	15 (31.3)	27 (56.3)	
	PhD	6 (1.5)	0 (0)	0 (0)	6 (100)	
Years of experience	< 1 year	128 (31.8)	19 (14.8)	44 (34.4)	65 (50.8)	0.695
	1- < 5 years	120 (29.9)	15 (12.5)	33 (27.5)	72 (60)	
	5-10 years	50 (12.4)	8 (16)	13 (26)	29 (58)	
	> 10 years	104 (25.9)	12 (11.5)	28 (26.9)	64 (61.5)	
Pharmacists’ working hours per week	24 hours or less	124 (30.8)	13 (10.5)	46 (37.1)	65 (52.4)	0.113
	24-40 hours	174 (43.3)	27 (15.5)	40 (23)	107 (61.5)	
	>40 hours	104 (25.9)	14 (13.5)	32 (30.8)	58 (55.8)	
Pharmacy location	City	297 (73.9)	37 (12.5)	88 (29.6)	172 (57.9)	0.49
	Village	95 (23.6)	16 (16.8)	25 (26.3)	54 (56.8)	
	Camp	10 (2.5)	1 (10)	5 (50)	4 (40)	
Pharmacy opening hours/week	<80 h	130 (32.3)	15 (11.5)	41 (31.5)	74 (56.9)	0.526
	80-120 h	213 (53.0)	32 (15)	64 (30)	117 (54.9)	
	7 days-24/7	59 (14.7)	7 (11.9)	13 (22)	39 (66.1)	

*Statistically significant.

Most participant characteristics were not significantly associated with knowledge scores (P-value > 0.05). However, pharmacists’ professional profile was significantly associated with knowledge score (P-value = 0.014): pharmacy managers showed the highest proportion of knowledgeable respondents (73.5%) in comparison with staff pharmacists (56.7%) and pharmacy owners (38.2%).

### Asthma knowledge assessment

#### General asthma knowledge.

Table 3 presents responses to items assessing pharmacists’ general asthma knowledge. Knowledge was highest for foundational concepts; most participants recognized asthma as a multifactorial disease (96.5%) and identified typical respiratory symptoms (94.0%). High proportions also acknowledged common triggers and the role of poor inhaler technique and non-adherence in poor asthma control (>85% for these items). In contrast, fewer pharmacists reported knowing how to use a peak flow meter (59.9%) or how to assess asthma severity (63.7%), and 64.7% reported awareness of recent asthma treatment guidelines.

**Table 3:** Pharmacists’ general knowledge about asthma.

Item	Yes N (%)	No N (%)
Asthma results from complex interactions between genetic, immunological, and environmental factors.	**388 (96.5)**	14 (3.5)
Do you know the typical respiratory symptoms of asthma?	**378 (94)**	24 (6)
Do you know how to use the peak flow meter?	**241 (59.9)**	161 (40.1)
Do you know how to assess the severity of your asthma patient?	**256 (63.7)**	146 (36.3)
Are you aware of the recent asthma treatment guidelines?	**260 (64.7)**	142 (35.3)
Do you know that patients should avoid spirometry with confirmed or suspected COVID-19 cases?*	**254 (63.2)**	148 (36.8)
Using steroid inhalation can significantly affect a child’s growth.	**284 (70.6)**	118 (29.4)
Asthma treatment guidelines no longer recommend SABA treatment alone. It does not recommend the use of SABA as a first choice; it recommends using long-acting beta2-agonists (formoterol) and inhaled corticosteroids (budesonide) as the first choice reliever of symptoms in adults with asthma.	**324 (80.6)**	78 (19.4)
SABA-only therapy raises the danger of life-threatening exacerbations and deaths due to asthma.	**282 (70.1)**	120 (29.9)
To reduce the risk of infection transmission during epidemics, patients should avoid using nebulizers as much as possible.	**211 (52.5)**	191 (47.5)
Physiological factors (e.g., pneumonia, liver disease, viral infection) affect theophylline drug concentration.	**349 (86.8)**	53 (13.2)
Factors like poor technique and non-adherence lead to poor asthma control.	**373 (92.8)**	29 (7.2)
Asthma can be triggered by different factors (e.g., sinus infections, allergies, and GERD).	**342 (85.1)**	60 (14.9)
If one of the parents has asthma, the risk of asthma in the child increases.	**327 (81.3)**	75 (18.7)
Local side effects of inhaled steroids include oropharyngeal candidiasis, hoarseness (dysphonia), and cough due to upper respiratory irritation.	**364 (90.5)**	38 (9.5)
Asthma occurs in most children with food allergies, especially to cow’s milk, and/or atopic dermatitis, regardless of the severity of the disease.	**275 (68.4)**	127 (31.6)
The presence of food allergies in children is an important indicator to predict the severity of asthma.	**260 (64.7)**	142 (35.3)

COVID-19: Coronavirus disease 2019, GERD: Gastroesophageal reflux disease, SABA: Short-acting beta2-agonist. * This item was originally framed in the context of COVID-19; in this study, it is interpreted as reflecting general infection-prevention considerations relevant to respiratory outbreaks and aerosol-generating procedures.

**Fig 1:**
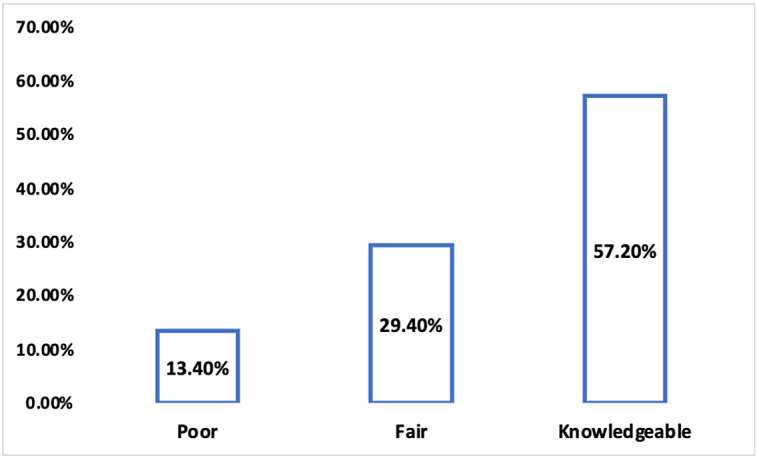
Level of overall knowledge score of asthma management.

Overall, 230 pharmacists (57.2%) were classified as knowledgeable, 118 (29.4%) had fair knowledge, and 54 (13.4%) had poor knowledge ‌‌(Fig 1).

#### Asthma pharmaceutical care knowledge.

Pharmacists’ agreement toward asthma pharmaceutical care knowledge statements are presented in Table 4. The mean agreement score for all items was 2.83 ± 0.26 on a 3-point Likert scale (1 = disagree, 2 = neutral, 3 = Agree).

**Table 4:** Pharmacists’ agreement toward asthma pharmaceutical care knowledge statements.

Item	Agree N (%)	Neutral N (%)	Disagree N (%)	Mean ± SD
The pharmacist plays an important role in the asthma care team.	327 (81.3)	72 (17.9)	3 (0.7)	2.81 ± 0.14
Asthma pharmaceutical care provided by community pharmacists results in improved clinical and economic outcomes.	326 (81.1)	71 (17.7)	5 (1.2)	2.80 ± 0.43
Because asthma treatment is highly variable, it should be monitored periodically.	344 (85.6)	55 (13.7)	3 (0.7)	2.85 ± 0.38
Asthma control is affected by many physiological, environmental, and behavioral factors.	361 (89.8)	39 (9.7)	2 (0.5)	2.89 ± 0.33
Monitoring therapeutic outcomes by community pharmacists is an effective strategy to improve the quality of medication therapy for asthma patients in primary care.	321 (79.9)	72 (17.9)	9 (2.2)	2.78 ± 0.47
The proper use of inhalers and inhalation techniques is one of the most important counselling aspects provided by pharmacists to patients.	360 (89.6)	39 (9.7)	3 (0.7)	2.89 ± 0.34
The provision of pharmaceutical care positively affects cost savings.	317 (78.9)	78 (19.4)	7 (1.7)	2.77 ± 0.46
It is important to evaluate the patient’s compliance with the medication, inhaler technique, and environmental control measures before prescribing a new medication.	349 (86.8)	49 (12.2)	4 (1.0)	2.86 ± 0.38
Using the wrong inhaler device causes more frequent emergency room and hospital admissions.	334 (83.1)	56 (13.9)	12 (3.0)	2.80 ± 0.47
Asthma pharmaceutical care knowledge (overall)				2.83 ± 0.26

### Pharmacist attitudes and behaviors

Pharmacists’ attitudes and behaviors toward asthma care are presented in Table 5. The average attitude score for all items was 3.97 ± 0.34 on a 5-point likert scale (1 = strongly disagree to 5 = Strongly Agree).

**Table 5:** Pharmacists’ attitudes and behaviors toward asthma care.

Item	Strongly agree N (%)	Agree N (%)	Neutral N (%)	Disagree N (%)	Strongly disagree N (%)	Mean ± SD
Pharmacist intervention has a positive impact on asthma-related outcomes in patients.	24 (6)	307 (76.4)	68 (16.9)	3 (0.7)	0(0)	3.92 ± 0.46
Asthma education provided by pharmacists is more effective than usual care in improving clinical outcomes.	44 (10.9)	309 (76.9)	41 (10.2)	8 (2)	0(0)	3.88 ± 0.49
Community pharmacists are required to receive continuing professional training to update their knowledge and skills.	30 (7.5)	306 (76.1)	58 (14.4)	8 (2)	0(0)	3.97 ± 0.54
Asthma training offered to pharmacists should be on asthma self-management.	30 (7.5)	325 (80.8)	43 (10.7)	4 (1)	0 (0)	3.89 ± 0.54
Pharmacist review of patients’ asthma medication treatment results in a decrease in the average frequency of acute attacks.	104 (25.9)	211 (52.5)	76 (18.9)	10 (2.5)	1 (0.2)	3.95 ± 0.47
The outcome of asthma treatment depends more on the patient’s behavior than on the efforts of healthcare providers.	39 (9.7)	325 (80.8)	35 (8.7)	3 (0.7)	0 (0)	4.01 ± 0.76
Successful management of asthma requires good communication between the patient and the healthcare team.	37 (9.2)	355 (88.3)	6 (1.5)	4 (1)	0 (0)	4.00 ± 0.46
Communication between healthcare professionals and patients should be improved to prevent suboptimal medication use in asthma patients.	38 (9.5)	327 (81.3)	33 (8.2)	4 (1)	0 (0)	4.06 ± 0.38
Lack of proper asthma education can be a big cause of incorrect device use.	41 (10.2)	321 (79.9)	36 (9)	4 (1)	0 (0)	3.99 ± 0.47
Inhaler devices should be prescribed after providing the necessary training on the use of the device and ensuring that the patient can use this device.	103 (25.6)	217 (54)	75 (18.7)	6 (1.5)	1 (0.2)	3.99 ± 0.48
The technique of using asthma medications such as Turbuhaler should be demonstrated to the patient by pharmacists.	40 (10)	324 (80.6)	31 (7.7)	7 (1.7)	0 (0)	4.03 ± 0.72
There is a need for patient education about asthma in Palestine	24 (6)	307 (76.4)	68 (16.9)	3 (0.7)	0 (0)	3.99 ± 0.50
Pharmacists’ attitudes and behaviors (overall)						3.97 ± 0.34

### Education and training for asthma management

As shown in Table 6, only 42.5% reported that their undergraduate training in asthma management was enough, while only 34.3% reported receiving post-graduation asthma training and education.

**Table 6:** Undergraduate and postgraduate pharmacists’ education perception.

Item	Yes N (%)	No N (%)	Not sure N (%)
Do you think that you have received adequate training on asthma management during the course of your university study?	171 (42.5)	218 (54.2)	13 (3.2)
Have you ever received any specific education on asthma management (post-graduation)?	138 (34.3)	211 (52.5)	53 (13.2)

### Sources of asthma information

**Fig 2:**
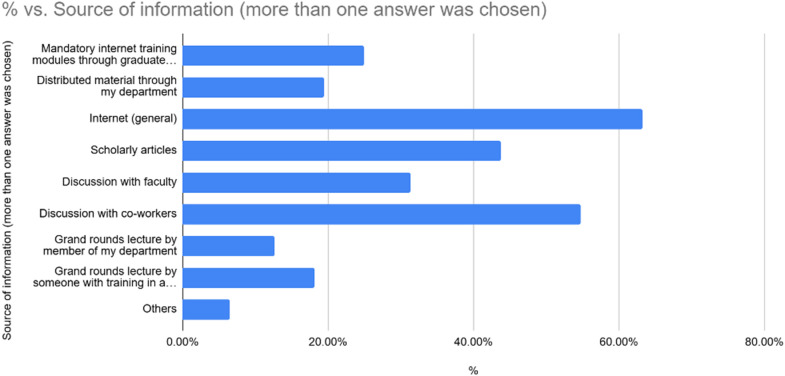
Sources of pharmacists’ information about asthma.

Participants indicated various sources of information for asthma management, as shown in Fig 1, with the most commonly referenced being general internet resources (63.2%) and discussions with colleagues (54.7%) (Fig 2).

### Frequency of exposure to asthma patients

As shown in Table 7, the most commonly reported frequency of exposure to asthma patients was two to three days per week (27.4%), followed by once per week (20.4%) and less than once per month (19.4%).

**Table 7:** Pharmacists’ frequency of exposure to asthma patients.

Frequency	N (%)
Less than once a month	78 (19.4)
Once a month	63 (15.7)
Once a week	82 (20.4)
Two to three days a week	110 (27.4)
Four or more days a week	60 (14.9)
Other	9 (2.2)

### Pharmacist practice towards asthma management

Table 8 summarizes pharmacists’ reported practices related to asthma management. The most frequently reported practices were checking inhaler technique (83.6%), teaching self-monitoring of symptoms (82.3%), and identifying modifiable risk factors for poor outcomes (80.8%). In contrast, 52.5% reported checking whether patients had a written asthma action plan.

**Table 8:** Pharmacists’ practices toward asthma management.

Item	Yes N (%)	No N (%)
Do you identify the modifiable risk factors for poor asthma outcomes?	**325 (80.8)**	77 (19.2)
Do you check if the patient has a written asthma plan?	**211 (52.5)**	191 (47.5)
Do you check patients’ inhalation technique?	**336 (83.6)**	66 (16.4)
Do you ask patients about their preference in asthma treatment?	**253 (62.9)**	149 (37.1)
Do you ask the patient about their treatment side effects?	**313 (77.9)**	89 (22.1)
Do you open an empathic discussion with patients about their adherence?	**281 (69.9)**	121 (30.1)
Do you advise patients to regularly take their ICS, as that might worsen their asthma medications?	**281 (69.9)**	121 (30.1)
Do you advise patients to discuss with you before stopping any of their medication?	**317 (78.9)**	85 (21.1)
Do you teach patients about self-monitoring of symptoms?	**331 (82.3)**	71 (17.7)

### Pharmacists’ barriers towards asthma management

Table 9 summarizes pharmacists’ reported barriers to providing asthma management services. The most frequently reported barriers were patients’ perception that asthma follow-up is not the pharmacist’s role (66.9%), limited patient time (65.2%), limited pharmacist time (61.4%), and lack of an asthma action plan (61.7%).

**Table 9:** Pharmacists’ barriers towards asthma management.

Item	Yes N (%)
Pharmacist does not have time	247 (61.4)
Patient does not have time	262 (65.2)
No financial incentive	215 (53.5)
I do not think that following asthma patient is my responsibility	141 (35.1)
I do not have enough knowledge	202 (50.2)
I do not have an asthma action plan	248 (61.7)
Patient’s perception that it is not the pharmacist’s role	269 (66.9)

### Factors associated with knowledge, pharmaceutical care knowledge, and attitudes

The study also performed multivariable regression analyses to examine the relationship between pharmacists’ characteristics and their knowledge, care plan knowledge, attitudes, and behaviors; the results are presented in Table 10. Overall, the regression models demonstrated very limited explanatory power (low R² values) and were not statistically significant at the model level (overall F-tests p > 0.05), indicating that the included predictors explained only a small proportion of the variance in domain scores.

**Table 10:** Multivariable regression analysis between pharmacists’ characteristics and pharmacists’ knowledge, care plan knowledge, attitudes, and behaviors.

Variable	Pharmacists’ Knowledge		Asthma pharmaceutical care knowledge		Attitudes and behaviors	
	Beta	P-value	Beta	P-value	Beta	P-value
Gender	−.049-	.381	.040	.477	.023	.680
Age (years)	−.055	.534	.010	.913	.063	.484
Profile	−.085	.144	.036	.538	.021	.720
Working settings	.110	.032*	−.093	.070	−.038	.458
Educational level	.074	.179	.000	.994	.051	.364
Years of experience	.044	.641	.123	.200	−.002	.986
Pharmacists’ working hours per week	.057	.348	.009	.887	−.006	.916
Geographic location of the pharmacy	−.003	.949	−.008	.869	.085	.098
Province	.107	.915	−.075	.145	−.011	.829
Number of hours/week pharmacy is open	.775	.439	−.003	.952	−.014	.785
Model	Dependent variable: pharmacist’s knowledge, R-square = 0.037, F = 1.481, P-value = 0.144		Dependent variable: Asthma pharmaceutical care plan knowledge R-square = 0.027, F = 1.078, P-value = 0.378		Dependent variable: attitudes and behaviors, R-square = 0.014, F = 0.555, P-value = 0.850	

* A P-value < 0.05 is considered statistically significant.

Within this context, working setting was associated with general asthma knowledge (standardized β = 0.110, p = 0.032), the overall model was not statistically significant and demonstrated low explanatory power; therefore, this association should be interpreted cautiously as exploratory and not indicative of a reliable predictive relationship.

Sensitivity analysis using Poisson regression based on the original count score yielded similar findings, with no substantial changes in the direction or significance of associations.

Furthermore, no statistically significant associations were identified between pharmacists’ characteristics and asthma pharmaceutical care knowledge, and no significant associations were observed for attitudes/behaviors (all p > 0.05).

## Discussion

In this study of pharmacists in Palestine, more than half of the study participants (57.2%) were classified as knowledgeable regarding asthma management. Knowledge was highest for basic concepts (e.g., recognition of asthma as a multifactorial disease (96.5%) and identification of typical respiratory symptoms (94.0%)), but gaps were observed in awareness of recent guideline updates and in topics related to infection-control practices during outbreaks. These findings suggest that while pharmacists are comfortable with core asthma concepts, the translation of updated recommendations into routine practice may remain suboptimal.

Our results align with previous studies performed in Turkey [1] and Al-Khartoum [10] reporting generally adequate baseline asthma knowledge among pharmacists, alongside persistent gaps in guideline awareness. For example, studies from Jordan have similarly shown that only 33.9% of pharmacists know about the need to reduce nebulizer use during epidemics to reduce the risk of infection transmission. This gap may reflect limited awareness of infection-prevention practices related to aerosol-generating procedures during respiratory outbreaks. Although originally framed in the context of COVID-19, this item is interpreted more broadly as reflecting preparedness for infection control in respiratory care settings [12]. Beyond individual knowledge gaps, these differences may also reflect variation in health system preparedness and dissemination of updated clinical guidance.

Additionally, another study conducted in Jordan has shown that 84% and 86% of study participants, the majority of whom are pharmacists, have high knowledge of the nature and symptoms of asthma [13]. However, only 47% of participants were aware of the recent changes in the GINA guidelines [13]. Another study performed in Egypt has also found that only 33.3% were aware of the latest GINA guidelines [7]. Taken together, these findings support the need for structured continuing professional development (CPD) and regionally tailored asthma education programs to improve uptake of guideline updates. Accordingly, structured CPD may help support pharmacists in applying updated guideline recommendations in practice. Furthermore, asthma education programs need to be developed in the region as recommended by prior work in Egypt, which also found a gap in this area [11].

It has been shown in this study that knowledge level differed by professional profile in bivariate analyses, and working setting was associated with knowledge score in multivariable analysis; however, the overall regression model demonstrated low explanatory power and was not statistically significant, indicating that these associations should be interpreted cautiously as exploratory rather than predictive. This finding is consistent with studies from Egypt and Turkey showing mixed results regarding differences between community and hospital pharmacists [7,14]. Such variability may reflect differences in pharmacists’ roles, scope of practice, and integration into clinical care across healthcare systems.

These findings highlight that pharmacist knowledge may be influenced more by structural and system-level factors, such as access to training, clinical role expectations, and regulatory frameworks, than by individual demographic characteristics alone.

Differences between our findings and those reported in studies from countries such as Jordan and Egypt may be partially explained by variations in pharmacy practice regulations and the scope of pharmacists’ roles across healthcare systems. In some settings, pharmacists are more formally integrated into clinical care and chronic disease management, supported by structured guidelines, reimbursement models, and mandatory continuing professional development programs. In contrast, variability in regulatory frameworks and practice expectations may limit pharmacists’ involvement in direct patient care, contributing to gaps in knowledge translation and practice. These findings suggest that improving asthma management may require not only individual-level educational interventions but also system-level changes, including strengthening regulatory support, expanding pharmacists’ clinical roles, and enhancing access to structured training programs.

Regarding asthma pharmaceutical care perceptions, the majority of study participants reported high agreement on the vital role of pharmacists in asthma management and the success of treatment regimens. 81.1% of pharmacists agreed that asthma pharmaceutical care provided by community pharmacists results in improved clinical and economic outcomes. However, the consistently high agreement across attitudes/behaviours items may reflect social desirability bias and ceiling effects; therefore, these findings may overestimate actual clinical behaviour and should be interpreted cautiously.

Additionally, a significant portion of the participants supports the importance of proper inhaler technique as an essential component of asthma pharmaceutical services offered by pharmacists. Evidence from a randomized controlled trial in community pharmacies in Portugal suggests that pharmacist-led inhaler technique interventions can improve technique and reduce healthcare utilization [15]. However, our findings are observational and self-reported and therefore do not allow inference about intervention effectiveness in this setting.

The pharmacist’s attitude towards asthma pharmaceutical care is important because, without a positive attitude, even adequate knowledge will not be utilized effectively [11]. In this study, positive attitudes towards asthma care were observed among pharmacists, 25.9% of pharmacists strongly agree that the pharmacist review of patients’ asthma medications leads to a decrease in the average frequency of acute attacks. As pharmacists are often the final healthcare contact before medication dispensing, they may be well-positioned to assess regimen appropriateness and provide counseling; however, the extent to which these activities occur in routine practice requires objective verification [1].

The pharmacist is considered an integral part of asthma management due to the higher frequency of asthma patients encountered each day. In our study, more than a quarter of the participants (27.4%) said they saw asthmatic patients two to three times a week. These findings emphasize the importance of continuous education for pharmacists on this topic; however, it has been observed that only 34.3% received any specific education on asthma management after graduation, indicating that post-graduation training in asthma is inadequate among pharmacists. Continuing professional development may help strengthen pharmacists’ confidence and consistency in delivering evidence-based asthma services and maintaining updated knowledge [16].

Regarding the sources of information for asthma that pharmacists generally use, in this study, the most commonly referenced were general internet resources (63.2%) and discussions with colleagues (54.7%). These findings are consistent with a study conducted in Jordan, which has found that the main source of information for asthma management was the internet, with 80.3% reporting it as their primary source [12]. This reliance on non-curated sources may partly contribute to gaps in awareness of guideline updates and supports the need for accessible, evidence-based resources.

In relation to pharmacists’ practices, the least frequently performed activities included initiating discussions about adherence, advising patients on the regular use of inhaled corticosteroids, exploring patients’ preferences regarding asthma treatment, and assessing whether patients possess a written asthma action plan. Among these, the assessment of a written asthma action plan was the least commonly reported practice, highlighting a substantial gap in patient-centered asthma care. Notably, only 52.5% of pharmacists reported checking written asthma action plans, representing a major practice gap in structured self-management support, which is a cornerstone of guideline-based asthma care. This finding highlights the immediate necessity for focused interventions and training to enhance pharmacists’ involvement in these critical aspects of asthma management. In addition, system supports (e.g., workflow time, access to standardized action plan templates, and patient education tools) may be required to enable consistent implementation.

In our study, the primary obstacles identified for effective asthma management included a lack of time for both the pharmacist (61.4%) and the patient (65.2%), the patient’s belief that asthma management is not the pharmacist’s responsibility (66.9%), and the absence of a reliable asthma action plan involving the pharmacist (61.7%). These findings were consistent with a study conducted in Jordan, which found a lack of time by the patient and unavailability of an asthma action plan were the most common barriers. However, they identified the lack of financial incentive as one of the three most common barriers to asthma management [17].

### Limitations

Several limitations should be considered when interpreting these findings. First, the study used a non-probability convenience/volunteer-response online sampling approach, which may introduce selection bias and limits generalizability to all pharmacists in Palestine; notably, younger pharmacists were over-represented (59% aged 20–29), which may reflect the online recruitment method and differential participation. Second, outcomes were measured using self-administered, self-reported questionnaires; therefore, responses may be influenced by recall error and may not fully reflect actual clinical practice. In particular, the consistently high agreement observed in attitudes/behaviours items suggests potential social desirability bias and ceiling effects, which could overestimate true attitudes or readiness to provide services. Third, reported practices represent perceived or stated behaviors rather than objectively observed practice, and future studies using direct observation, audit, or intervention designs are needed to confirm whether these self-reported practices translate into measurable improvements in patient care. Some knowledge items were framed in the context of COVID-19 due to the timing of questionnaire development. Although these items were interpreted as reflecting general infection-prevention principles, their time-specific wording may limit generalizability to future outbreaks with different clinical guidance.

Fifth, one item in the general asthma knowledge section (using inhaled corticosteroids significantly affects children’s growth) represents a simplified statement that does not fully reflect the evidence, which indicates that growth effects are dose-dependent and influenced by treatment duration and patient-specific factors. This oversimplification may have introduced misclassification bias in the assessment of pharmacists’ knowledge and reflects a limitation in the construct validity of the measurement tool. The question was simplified and did not fully address that this issue is dose- and context-dependent. Therefore, misclassification of the pharmacists’ knowledge may have occurred. Finally, the cross-sectional design precludes causal inference and limits assessment of temporality.

## Conclusion

This study suggests that Palestinian pharmacists have acceptable basic asthma knowledge and positive attitudes, but clinically important gaps persist in areas aligned with recent guideline-based care and in delivering structured self-management support. However, these findings are based on a cross-sectional, self-reported survey and should be interpreted cautiously, particularly given the very limited explanatory power of the regression models.

Accordingly, the findings identify priority areas for improvement, including continuing professional development focused on guideline updates and practical counseling skills (e.g., inhaler technique assessment and action plan review). Future studies using objective assessments and/or intervention designs are needed to determine whether such strategies translate into measurable improvements in pharmacists’ practice and patient outcomes. System-level supports (e.g., protected time and access to care tools) may also be required to address reported barriers.

## Supporting information

S1 File Survey on KAP regarding Asthma care among pharmacists. (DOCX)

S1 Table Sociodemographic characteristics by knowledge score. (DOCX)

S1 Dataset De-identified participant-level dataset underlying the findings of the study (Excel format). (XLSX)
