# Supplementary material for: Knowledge, attitudes, and practices regarding asthma management among pharmacists in palestine: A cross-sectional study
Source: PLoS One. 2026 Jun 18;21(6):e0351933. doi: 10.1371/journal.pone.0351933 (PMC13278420; doi:10.1371/journal.pone.0351933)
Supplement: S1 File — (DOCX) [file pone.0351933.s001.docx]

# **Factors Influencing Knowledge, Attitudes, and Practices Relating Asthma Management Among Pharmacists: A Cross-Sectional Study from Palestine.**

**Introduction to questionnaire:**

**You are being invited to take part in a web-based survey examining the determinants of pharmacists' willingness to become potential immunizers of adults. Your contribution to this study is valuable and create a better future for healthcare system in our community by sharing your experiences and viewpoints.**

**This questionnaire should just take a few minutes to complete because it is meant to be simple and considerate of your time. Please be assured that we will maintain confidentiality of your answers and no personal information will be shared. The research team will be the only party to review the results.**

***The target group is: All pharmacists with practicing certificate**

# **Section one: Sociodemographic characteristics:**

| Category | Options |
| --- | --- |
| 1. Gender | ☐ Male  ☐ Female |
| 1. Age (years) | ☐ 20–29  ☐ 30–39  ☐ 40–49  ☐ ≥50 |
| 1. Profile | ☐ Manager  ☐ Owner |
|  | ☐ Staff Pharmacist |
| 1. Working settings | ☐ Community pharmacies  ☐ Hospital pharmacies outpatient  ☐ Hospital pharmacies inpatient |
| 1. Educational Level | ☐ Bachelor of Pharmacy  ☐ Pharm D  ☐ Master  ☐ PhD |
| 1. Years of Experience | ☐ less than one year  ☐ 1- 5  ☐ 5–10  ☐ More than 10 years |
| 1. Pharmacist’s Working Hours per Week | ☐ 24 hr or less  ☐ 25–40 hr  ☐ More than 40 hr |
| 1. Geographic Location of the Pharmacy | ☐ City  ☐ Village  ☐ Camp |
| 1. Province | ☐ Al Quds  ☐ Jenin  ☐ Nablus  ☐Tulkarem  ☐ Ramallah  ☐Beitlahem  ☐ Hebron  ☐ Jericho  ☐ Tubas  ☐ Salfit  ☐Qalqilia |
| 1. Number of Hours/Week Pharmacy is Open | ☐ Less than 80 h  ☐ 80–120 h  ☐ 7 days 24/24 |
| 1. Number of handled prescriptions per day |  |
| 1. Total number of employees at the practice site |  |

# **section two: Pharmacists’ knowledge:**

|  | Correct | Incorrect |
| --- | --- | --- |
| 1. Do you know the typical respiratory symptoms of asthma |  |  |
| 1. Do you know how to use the peak fow meter |  |  |
| 1. Do you how to assess severity of your asthma patient? |  |  |
| 1. Are you aware of the recent asthma treatment guidelines |  |  |
| 1. Do you know that patients should avoid spirometry with confrmed or suspected COVID-19 cases |  |  |
| 1. Using Steroid inhalation can significantly affect child’s growth |  |  |
| 1. Asthma treatment guidelines no longer recommends short-acting beta2-agonist (SABA) treatment alone. It does not recommend the use of SABA as first choices, it recommends using long-acting beta2-agonist (formoterol) and inhaled corticosteroids (Budesonide) as first choice reliever of symptoms in asthma adults. |  |  |
| 1. SABA-only therapy raises the danger of life-threatening exacerbations and deaths due to asthma |  |  |
| 1. To reduce the risk of infection transmission during epidemics, patients should avoid using nebulizers as much as possible. |  |  |
| 1. Physiological factors (eg Pneumonia, liver disease, viral infection) affect theophylline drug concentration |  |  |
| 1. Factors like Poor technique, non-adherence lead to poor asthma control |  |  |
| 1. Asthma can be triggered by different factors (eg, Sinus infections, allergies and GERD) |  |  |
| 1. If one of parents has asthma the risk of the asthma in child increases |  |  |
| 1. Local side effect of inhaled steroids include oropharyngeal candidiasis, hoarseness (dysphonia), and cough due to upper respiratory irritation |  |  |
| 1. Asthma occurs in most children with food allergies and/ or atopic dermatitis, especially cow’s milk, regardless of the severity of the disease. |  |  |
| 1. The presence of food allergy in children is an important indicator to predict the severity of asthma |  |  |

# **section three: Asthma pharmaceutical care knowledge:**

|  | Agree | Neutral | Disagree |
| --- | --- | --- | --- |
| The pharmacist plays an important role in the asthma care team. |  |  |  |
| Asthma pharmaceutical care provided by community pharmacists results in improved clinical and economic outcomes. |  |  |  |
| Because asthma treatment is highly variable, it should be monitored periodically. |  |  |  |
| Asthma control is affected by many physiological, environmental, and behavioural factors. |  |  |  |
| Monitoring therapeutic outcomes by community pharmacists is an effective strategy to improve the quality of medication therapy for asthma patients in primary care. |  |  |  |
| The proper use of inhalers and inhalation techniques is one of the most important counselling aspects provided by pharmacists to patients. |  |  |  |
| The provision of pharmaceutical care positively affects cost savings. |  |  |  |
| Studies investigating facilitators and barriers to asthma management are needed. |  |  |  |
| The most important member of the asthma care team is the patient. |  |  |  |
| It is important to evaluate the patient’s compliance with the medication, inhaler technique, and environmental control measures before prescribing a new medication in the treatment of asthma. |  |  |  |
| A significant portion of patients have difficulty using metered dose inhaler delivery systems. |  |  |  |
| Using the wrong inhaler device causes more frequent emergency room and hospital admissions. |  |  |  |
| Asthma causes individuals to lose their school and workdays. |  |  |  |
| The effect of asthma on mood is insignificant. |  |  |  |
| There is a significant relationship between asthma control level and gender. |  |  |  |
| Younger patients under 20 years of age have better asthma control than older patients. |  |  |  |
| Asthma patients do not benefit from disease monitoring with a peak flow meter. |  |  |  |
| Control of respiratory function in asthma care is very complex |  |  |  |

# **section four: Pharmacist attitudes and behaviors:**

|  | Strongly Agree | Agree | Neutral | Disagree | Strongly disagree |
| --- | --- | --- | --- | --- | --- |
| Pharmacist intervention has a positive impact on asthma-related outcomes in patients. |  |  |  |  |  |
| Asthma education provided by pharmacists is more effective than usual care in improving clinical outcomes. |  |  |  |  |  |
| Community pharmacists are required to receive continuing professional training to update their knowledge and skills. |  |  |  |  |  |
| Asthma training offered to pharmacists should be on asthma self-management. |  |  |  |  |  |
| Pharmacist review of patients’ asthma medication treatment results in a decrease in the average frequency of acute attacks. |  |  |  |  |  |
| The outcome of asthma treatment depends more on the patient’s behaviour than on the efforts of healthcare providers. |  |  |  |  |  |
| Successful management of asthma requires good communication between the patient and the healthcare team. |  |  |  |  |  |
| Communication between healthcare professionals and patients should be improved to prevent suboptimal medication use in asthma patients. |  |  |  |  |  |
| Lack of proper asthma education can be a big cause of incorrect device use. |  |  |  |  |  |
| Inhaler devices should be prescribed after providing the necessary training on the use of the device and ensuring that the patient can use this device. |  |  |  |  |  |
| The technique of using asthma medications such as Turbuhaler should be demonstrated to the patient by pharmacists. |  |  |  |  |  |
| There is a need for patient education about asthma in Palestine |  |  |  |  |  |

# **section five: Sources of information for asthma management:**

**1- Have you ever received any specific education on asthma management?**

❑ Yes

❑ No

❑ Not sure

| 2-Through which of the following have you received education regarding asthma management? (You may choose more than one answer) | | |
| --- | --- | --- |
| Mandatory internet training modules through Graduate pharmacist Education | **❑** | |
| Distributed material through my department | **❑** | |
| Internet (general) | **❑** | |
| Scholarly articles | **❑** | |
| Discussion with faculty | **❑** | |
| Discussion with co-workers | **❑** | |
| Grand rounds lecture by member of my department | **❑** | |
| Grand rounds lecture by someone with training in asthma management | **❑** | |
| Other (please specify)__________________ |  | |
| 3-Do you think that you have received adequate training on asthma management during the course of your university study? If not, please free text ideas for improved training opportunities. | | |
| Yes | | **❑** |
| No | | **❑** |
| Comments:_____________________ | |  |
| 4-How often are you with asthmatic patients through your work? | |  |
| Less than once a month | | **❑** |
| Once a month | | **❑** |
| Once a week | | **❑** |
| Two to three days a week | **❑** | |
| Four or more days a week | **❑** | |
| Other (please specify)__________________ |  | |

# **Section six: personal use:**

|  | Yes | No |
| --- | --- | --- |
| Do you perform a detailed history examination for asthma |  |  |
| Do you identify the modifiable risk factors for poor asthma outcomes? |  |  |
| Do you check if the patient has a written asthma plan? |  |  |
| Do you check patients inhalation technique |  |  |
| Do you ask patients about their preference in asthma treatment |  |  |
| Do you ask the patient about their treatment side effects? |  |  |
| Do you open an empathic discussion with patients about their adherence |  |  |
| Do you advise patients to regularly take their ICS as that might worsen their asthma medications |  |  |
| Do you advise patients to discuss with you before stopping any of their medication |  |  |
| Do you teach patients about self-monitoring of symptoms |  |  |
| Do you assess symptoms control over the last 4 weeks? |  |  |
| Do you use PEFM for follow-up of asthma patient? |  |  |
| Do you consider stepping down asthma treatment after proper asthma |  |  |
| Do you schedule a follow-up visit for asthma patients control for 3 months |  |  |

# **section seven: Barriers:**

|  | Yes | No |
| --- | --- | --- |
| Lack of time by the pharmacist |  |  |
| Lack of time by the patient |  |  |
| Pharmacists’ perception that it is not their role |  |  |
| Patient’s perception that it is not the pharmacist’s role |  |  |
| No financial incentive |  |  |
| Lack of pharmacist confidence and skills in asthma management |  |  |
| Lack of pharmacist confidence and skills in asthma counselling |  |  |
| Lack of pharmacist confidence and skills in asthma monitoring |  |  |
| Patient does not have time |  |  |
| I do not think that following asthma patient is my responsibility |  |  |
| I do not have enough knowledge |  |  |
| I do not have an asthma action plan |  |  |
