# Supplementary material for: Knowledge, attitudes, and practices regarding asthma management among pharmacists in palestine: A cross-sectional study
Source: PLoS One. 2026 Jun 18;21(6):e0351933. doi: 10.1371/journal.pone.0351933 (PMC13278420; doi:10.1371/journal.pone.0351933)
Supplement: S1 Table — (DOCX) [file pone.0351933.s002.docx]

S1 Table: Sociodemographic by knowledge score

| Variable | Category | Total (%) | Poor | Fair  n (%) | Knowledgeable  n (%) | P-Value |
| --- | --- | --- | --- | --- | --- | --- |
| Province | Al-Quds | 240 (59.7) | 32 (13.3) | 66 (27.5) | 142 (59.2) | 0.062 |
|  | Jenin | 16 (4.0) | 3 (18.8) | 3 (18.8) | 10 (62.5) |  |
|  | Nablus | 21 (5.2) | 4 (19) | 8 (38.1) | 9 (42.9) |  |
|  | Tulkarem | 12 (3.0) | 1 (8.3) | 2 (16.7) | 9 (75) |  |
|  | Ramallah | 25 (6.2) | 1 (4) | 8 (32) | 16 (64) |  |
|  | Bethlehem | 24 (6.0) | 4 (16.7) | 8 (33.3) | 12 (50) |  |
|  | Hebron | 35 (8.7) | 5 (14.3) | 10 (28.6) | 20 (57.1) |  |
|  | Jericho | 5 (1.2) | 1 (20) | 1 (20) | 3 (60) |  |
|  | Tubas | 2 (0.5) | 0 (0) | 2 (100) | 0 (0) |  |
|  | Salfit | 4 (1.0) | 3 (75) | 0 (0) | 1 (25) |  |
|  | Qalqilya | 3 (0.7) | 0 (0) | 2 (66.7) | 1 (33.3) |  |
|  | Gaza and others | 15 (3.7) | 0 (0) | 8 (53.3) | 7 (46.7) |  |
